# Digital Assessment of Wellbeing in New Parents (DAWN-P): protocol of a randomised feasibility trial comparing digital screening for maternal postnatal depression with usual care screening

**DOI:** 10.1186/s40814-025-01631-7

**Published:** 2025-04-12

**Authors:** Emily Eisner, Ria Agass, Elizabeth Camacho, Adedamola Falana, Mark Hann, Anulika Ifezue, Henna Lemetyinen, Holly Myers, Charlotte Stockton-Powdrell, Clare Tower, Kylie Watson, Pauline Whelan, Anja Wittkowski, Shôn Lewis

**Affiliations:** 1 Division of Psychology and Mental Health, School of Health Sciences, Faculty of Biology, Medicine and Health, University of Manchester, Manchester, UK; 2 Greater Manchester Mental Health NHS Foundation Trust, Manchester, UK; 3 NIHR Manchester Biomedical Research Centre, Manchester, UK; 4 Mersey and West Lancashire Teaching Hospitals NHS Trust, Prescot, UK; 5 Institute of Population Health, University of Liverpool, Liverpool, UK; 6 Centre for Biostatistics, Division of Population Health, Health Services Research and Primary Care, School of Health Sciences, Faculty of Biology, Medicine and Health, University of Manchester, Manchester, UK; 7 Manchester University NHS Foundation Trust, Manchester, UK; 8 Division of Informatics, Imaging and Data Sciences, Faculty of Biology, Medicine and Health, Centre for Health Informatics, University of Manchester, Manchester, UK; 9 Division of Nursing, Midwifery and Social Work, School of Health Sciences, Faculty of Biology, Medicine and Health, University of Manchester, Manchester, UK

**Keywords:** Postnatal depression, Digital mental health, mHealth, Smartphone, Maternal mental health, Screening

## Abstract

**Background:**

Meta-analyses indicate that 17% of mothers experience postnatal depression (PND) in the year following childbirth, with suicide the leading cause of direct maternal death between 6 weeks and 12 months postpartum. The consequences and costs of PND are particularly high due to impacts on infants as well as parents. If detected, PND usually responds well to psychological treatment and/or medication but national reports indicate > 50% of cases are undetected. To improve detection, we developed a digital screening system (CareLoop PND) whereby mothers use an app to monitor their mood daily using a validated measure (Edinburgh Postnatal Depression Scale; EPDS), with real-time responses uploaded to a secure server. In this paper, we describe the protocol of a study to determine feasibility of delivering a full-scale RCT comparing digital screening with standard NHS practice.

**Methods:**

In this single-blind randomised feasibility trial, participants (*n* = 80) will be recruited during late pregnancy from two NHS maternity services and randomised (1:1) to receive CareLoop PND alongside their usual NHS care, or usual care alone. Those in the experimental arm will use the CareLoop PND app daily from ≥ 36 weeks’ pregnancy until 8 weeks postpartum. During this period, participants scoring above EPDS screening thresholds (via the app or standard care) will be assessed to confirm diagnosis. True positive PND cases identified by digital screening will be referred to services for support. A blinded researcher will conduct follow-up assessments using clinical and health economic measures at 8 weeks and 6 months postpartum. At 8 weeks postpartum, experimental arm participants will also provide qualitative and quantitative feedback exploring app usability, acceptability, and implementation. Feasibility of delivering a full-scale RCT will be evaluated using a priori criteria relating to app engagement, study retention and completion of candidate primary outcome measures.

**Conclusions:**

Digital screening could increase appropriate referral to perinatal mental health care. However, prior to roll out in NHS services, evidence of efficacy and cost-effectiveness is needed. The current study protocol will determine whether a full-scale RCT examining efficacy and cost-effectiveness is feasible and will inform the design of such a trial.

**Trial registration:**

Prospective ISRCTN registration (03/07/23): ISRCTN10781027; https://www.isrctn.com/ISRCTN10781027 .

**Supplementary Information:**

The online version contains supplementary material available at 10.1186/s40814-025-01631-7.

## Background

Perinatal mental health is a global mental health concern [1], with 1 in 5 women experiencing a change in their mental health during their pregnancy or within a year of giving birth [2]. Meta-analyses indicate that 17% of mothers develop postnatal depression (PND) during the year after their child’s birth [3, 4], with suicide the leading cause of direct maternal death from 6 weeks to 12 months postpartum [5]. Although diagnostically identical to depression at other times, the consequences and costs of PND are substantially greater due to adverse effects on infants as well as parents. PND can affect parental wellbeing, the parent-infant bond, the child’s development, and parents’ and children’s long-term physical and mental health [6, 7].

In England, the publicly funded health service (National Health Service; NHS) has identified increasing access to high quality perinatal mental health care as a key priority in its Long Term Plan [8]. For parents and their children, the lifelong consequences of not accessing such care are estimated to cost the NHS and social care £1.2 billion per 1-year birth cohort [9]. Although medication and talking therapy can be used to successfully treat PND, identifying who needs treatment is difficult. National reports [7, 9, 10] and our local audit data (unpublished) suggest that more than half of cases of PND currently go undetected and untreated. Our study seeks to improve detection of maternal PND, to allow appropriate referral to existing perinatal mental health services.

At present, there is no nationally implemented method for monitoring maternal mental health, although the Edinburgh Postnatal Depression Scale (EPDS) is widely used and has been recommended [11]. The EPDS is a 10-item screening measure, usually used in-person by health visitors, which detects PND with 81% sensitivity and 88% specificity [12]. The current UK National Institute for Health and Care Excellence (NICE) guidelines [11] recommend that health professionals consider using two initial screening questions (known as the Whooley questions) at each postnatal visit and, if symptoms are detected, consider administering the full EPDS. However, there are significant barriers to screening, including limited staff time during busy postnatal visits, lack of staff training around perinatal mental health, parental unwillingness to disclose difficulties during visits, and language barriers (e.g. 20% of Manchester adults have a main language other than English [13]). Given that more than half of cases of PND are missed [7, 9, 10], a practical, cost-effective solution is needed to improve PND detection, identify PND earlier, and enable earlier intervention.

Most (97%) people of childbearing age own a smartphone [14, 15], presenting a clear opportunity for a major advance in improving PND detection. We developed the CareLoop PND digital screening system [16], in which a smartphone app prompts parents to answer the ten EPDS questions daily and wirelessly uploads their real-time responses to a secure server. In our proof-of-concept study [16], we asked 15 mothers and 8 partners to use the app from ≥ 36 weeks pregnancy until 6 weeks postpartum. The 23 participants completed 67% of daily app-based assessments during the 6- to 12-week study period. Three mothers (20%) screened positive for PND during the study. Concurrent validity between daily app-reported and standard weekly paper EPDS was high. Mean follow-up questionnaire ratings (4.1 out of 5) suggested moderate to high app acceptability. In qualitative interviews, participants reported that the app helped them to understand their mood and to voice concerns more openly than an in-person assessment did. All participants found the app quick and easy to use; some gave suggestions to improve app engagement. Overall, the concept of digital screening was highly acceptable. This is consistent with findings from the wider digital health literature that people with common [17] and severe [18] mental health problems find smartphone apps acceptable for monitoring symptoms, alongside face-to-face care, and that digital tools may reduce stigma [19–22], aid understanding of mental health fluctuations [23–25], and facilitate help-seeking [26] in such groups.

The CareLoop PND digital screening system could transform existing screening and increase referral to perinatal mental health care, supporting the NHS Long Term Plan ambitions. However, prior to roll-out in the NHS, a full-scale Randomised Controlled Trial (RCT) is needed to determine whether adding digital screening to usual care is efficacious and cost-effective [27]. The current protocol describes a randomised feasibility trial which gathers information required to plan and deliver a future full-scale RCT evaluating the CareLoop PND digital screening system.

## Objectives

The primary objective of this feasibility trial is to examine the feasibility of delivering a full-scale, single-blind, RCT, within NHS services, comparing digital PND screening and standard practice. Feasibility will be assessed against three a priori criteria defining acceptable levels of (i) app engagement, (ii) retention, and (iii) data completeness. Secondary objectives are to examine participant recruitment, barriers to recruitment, the suitability of two candidate primary outcomes for a full-scale trial, the suitability of other proposed clinical and health economic measures, and the acceptability, usability and safety of the digital screening system.

## Methods

### Trial design

**Fig. 1:**
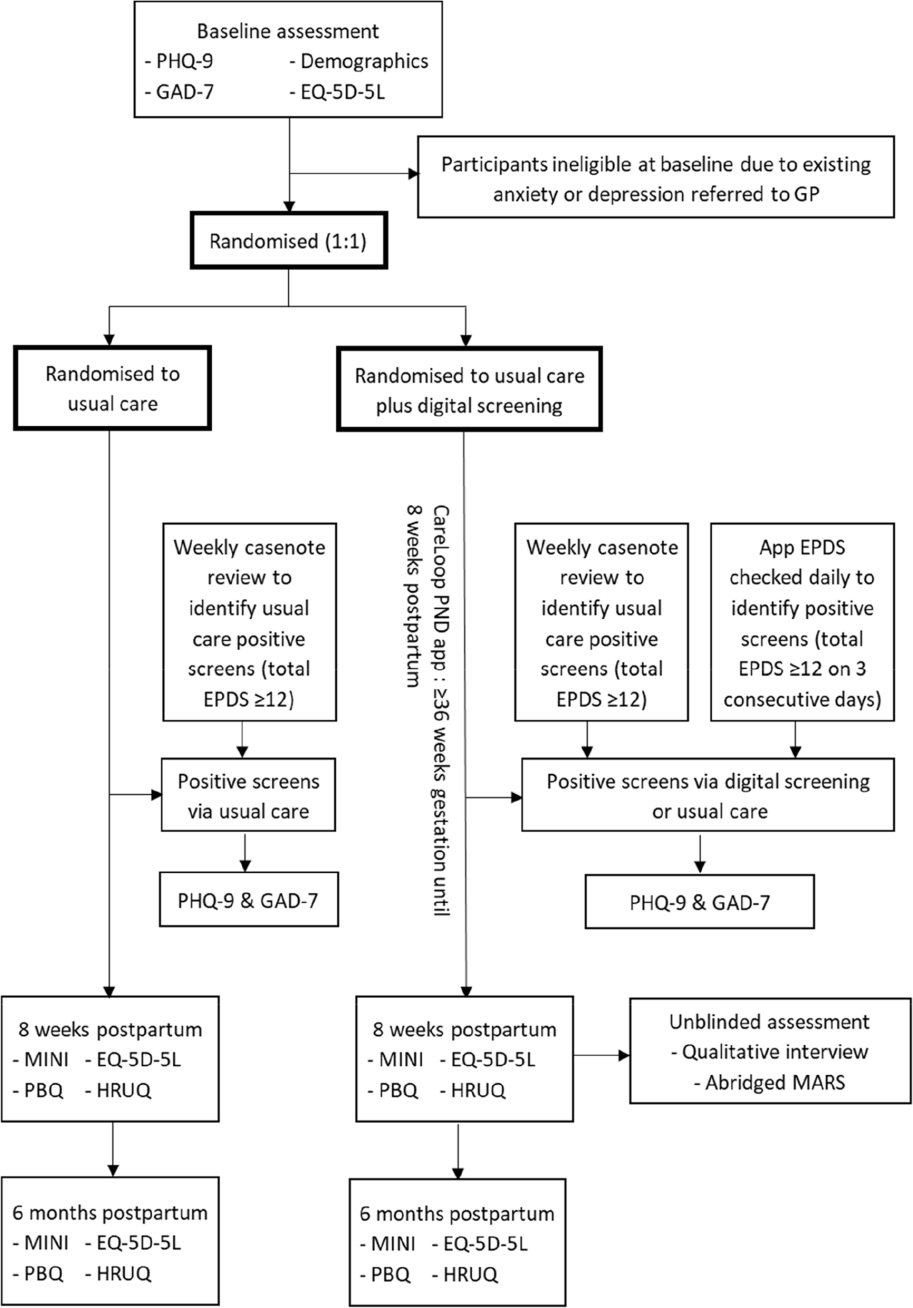
Study design and assessment schedule. PHQ-9 = Patient Health Questionnaire; GAD-7 = Generalised Anxiety Disorder questionnaire; EPDS = Edinburgh Postnatal Depression Scale; MINI = Mini International Neuropsychiatric Interview; PBQ = Postpartum Bonding Questionnaire; HRUQ = Healthcare Resource Use Questionnaire; Abridged MARS = Mobile App Rating Scale

A single blind, randomised feasibility trial (usual care plus digital screening vs. usual care alone; total *n* = 80), with measures administered pre-randomisation, post-intervention (8 weeks postpartum) and at later follow-up (6 months postpartum). See Fig. 1 for a flow diagram outlining the trial design.

### Ethical approval

This study was approved by Greater Manchester West Research Ethics Committee (approval number 23/NW/0064) and the UK Health Research Authority.

### Setting

The study sponsor is Greater Manchester Mental Health NHS Foundation Trust. The multi-site study will take place across two NHS sites: Manchester University NHS Foundation Trust (MFT) and Mersey and West Lancashire Teaching Hospitals NHS Trust (MWL; formerly named St Helens and Knowsley Teaching Hospitals NHS Trust).

### Participants

#### Sample size

The study aims to recruit 80 participants. A conventional power calculation is not necessary since evaluating intervention effectiveness is not the aim of this feasibility trial. The proposed sample size is sufficient to assess feasibility outcomes (e.g. to estimate the attrition rate to within ± 11%) and to estimate key parameters (e.g. the standard deviation of candidate primary outcomes) to help inform a power calculation for the larger trial [28]. With reference to the latter, as long as an upper, one-sided 80% confidence limit is used for the estimate of the standard deviation (rather than the estimated value itself), the larger trial has an 80% chance of achieving its planned power [29].

#### Eligibility criteria

Inclusion criteria.

Less than or equal to 36 weeks pregnant; under care of Manchester Foundation Trust (MFT) or Mersey and West Lancashire Teaching Hospitals NHS Trust (MWL); aged ≥ 18 years; sufficient English fluency to complete baseline assessment (or partner speaks English fluently).

Exclusion criteria.

Fetal abnormality; major depression (Patient Health Questionnaire- 9 (PHQ-9) score ≥ 10) [30], generalised anxiety disorder (Generalised Anxiety Disorder questionnaire- 7 (GAD-7) score ≥ 10) [31] or perinatal psychosis at study entry; stillbirth, pre-eclampsia or another medical emergency requiring hospital admission (participants experiencing these conditions in relation to childbirth during the study will be withdrawn); under the care of MFT but living outside the Central Manchester area; under the care of MWL but living outside the St Helens area.

Three of these eligibility criteria require further explanation.

- Inclusion from 36 weeks’ gestation. Although the specific period of interest for PND screening is *after* childbirth, participants will be recruited when still pregnant (≥ 36 weeks). This is to allow time for participants to complete key study procedures *before* giving birth, rather than in the immediate postnatal period when their focus will be on caring for their baby and recovering from childbirth. Including participants only after 36 weeks’ gestation is appropriate as the likelihood of unanticipated neonatal complications is considerably lower from this point onwards.
- Exclusion of stillbirth, pre-eclampsia or other medical emergencies requiring hospital admission. This exclusion criterion is designed to prioritise the mental and physical health of participants during the study. Senior clinical experts on our study team considered it inappropriate for participants experiencing these distressing, life-threatening, and/or life-changing conditions to continue participating in the study. For example, requiring a parent who has just experienced stillbirth to continue to participate in a study investigating their postnatal experience is likely to add to their distress. Given that participants will be randomised during late pregnancy and that the named conditions do not typically present until childbirth itself, it is necessary to allow participants to be excluded post-randomisation if these conditions arise.
- Exclusion of participants outside Central Manchester and St. Helens areas. These geographical restrictions are in place to enable the research team to access relevant electronic health records (GP and health visitor records), which are only readily accessible for people living in the Central Manchester (for MFT) or St Helens (for MWL) areas. Researchers will need to access these health records to extract specific data (true/false positive PND screens via usual care screening) that underpins one of the candidate primary outcomes for a full-scale study.

#### Recruitment

Participants will be recruited from maternity services at the two study sites. Health professionals (e.g. obstetricians, midwives, health visitors) will identify potential participants and provide them with initial study information. When potential participants are interested, the health professional will obtain verbal or written consent to pass on their contact details to a researcher, who will provide full study information prior to collecting formal consent. The study will also be publicised via social media (e.g. Twitter (X), Facebook posts) and posters in NHS services. Posters and social media adverts will include a QR code that will lead participants to a short video advertisement of the study. Participants can self-refer by contacting the research team directly.

#### Informed consent

A researcher will provide potential participants with a verbal explanation of the study, a participant information sheet, and a weblink to view a participant information video. They will answer any questions and provide further information as required to ensure fully informed consent. Individuals choosing to participate will be asked to provide formal audio-recorded consent. Eligible individuals declining to participate will be asked for brief reasons (optional). Participants ineligible due to existing anxiety/depression will be referred to their GP, midwife, or health visitor.

### Assignment of interventions

#### Allocation and sequence generation

Following baseline assessment (see “Data collection and management” section for assessment details), participants will be randomly allocated (1:1) using an online service (www.sealedenvelope.com) to one of two study groups: usual care plus digital screening, or usual care alone. Random allocation sequences in blocks of size 4 or 6 will be generated, with separate sequences generated for each study site (MFT, MWL) to maintain balanced treatment allocation. Block sizes occur with equal frequency and are randomly determined.

### Implementation

An unblinded member of the research team (e.g. project manager, research midwife) will input the participant’s ID number and study site into the online Sealed Envelope system. The system sends the random group allocation via email to the unblinded team member, who will inform the participant of their allocated study group and arrange the app onboarding session if applicable.

#### Blinding

The trial statistician and the research assistant conducting all follow-up assessments will be blinded to treatment allocation. The circumstances around any un-blindings will be recorded and discussed. Other research team members (e.g. project manager, research midwife) will not be blinded to treatment allocation as they will conduct study procedures requiring knowledge of participants’ treatment allocation (e.g. app setup, monitoring app responses). It will also not be possible to blind participants to the group of allocation.

### Intervention and control groups

#### Usual care plus digital screening

Participants in the digital screening group will be asked to use the CareLoop PND app from study entry until 8 weeks postpartum, in addition to their usual NHS care. This specific intervention end date was selected to enable a fair comparison with usual care: within UK health services, at 6–8 weeks postpartum, mothers should be offered a health check, including mental health screening questions [32]. Research midwives will instruct participants in how to download, configure and use the app, with the help of an instructional video. Participants without a suitable smartphone will be lent a study phone until 8 weeks postpartum. All participants will be offered £10 phone credit per month that they are using the app, up to £30 in total.

The CareLoop PND app will prompt participants daily (between 10 am and 5 pm) to answer the ten questions of the Edinburgh Postnatal Depression Scale (EPDS) [12], in their preferred language (English, Urdu, Arabic, Cantonese). Our previous study demonstrated that receiving prompts to complete EPDS daily was acceptable to participants and that there was high concurrent validity between daily app-reported and standard weekly paper EPDS [16]. Each question of the EPDS has a choice of 4 answers. To ensure convenience, participants can answer the questions at any point during the day. Participants’ EPDS responses will be automatically uploaded to a secure server. An unblinded researcher will review the responses daily and take specific actions if the total EPDS score or the EPDS self-harm item are above the following thresholds:

- Total EPDS. If the total EPDS score is ≥ 12 on three consecutive reports, this constitutes a positive PND screen (during periods where EPDS is not completed every day, the next completed report will be used). A blinded researcher will contact all participants screening positive within 48 h to conduct assessments of depression (Patient Health Questionnaire- 9; PHQ-9) [30] and anxiety (Generalised Anxiety Disorder- 7; GAD-7) [31]. The screening outcome will be classified as a true positive or false positive according to the assessments, and actions taken as follows:
  o True positives (PHQ-9 ≥ 10 or GAD-7 ≥ 10): an unblinded researcher will send the midwife or health visiting team symptom information via secure NHS email within 48 h.
  o False positives (PHQ-9 < 10 and GAD-7 < 10): no further action required.
- Self-harm. If the EPDS self-harm question (item 10) is rated > 1, an unblinded researcher will inform the participant’s midwife or health visiting team via secure NHS email within 24 h (weekdays) or on the next working day (weekend days). If the self-harm question is rated > 1 on a weekend day, an unblinded researcher will contact the participant to conduct a risk assessment and signpost to services as appropriate.

The app contains additional features, such as a graph of the user’s responses to the daily questions over time, randomly selected daily inspirational quotes, a diary for the user to keep their own daily notes about their mental health (not uploaded), phone numbers of local and national mental health helplines, and links to external resources with information about parenting and baby health. Research midwives will phone participants 1, 2, 4, and 8 weeks after randomisation to check for adverse events (reported in the study’s adverse events form) and/or technical issues (referred to the technical team). The timing of these phone calls has been chosen to roughly mirror the timing of antenatal and postnatal midwife/health visitor appointments in standard care.

#### Usual care alone

Participants allocated to this group will continue with their usual NHS care, which typically includes some mental health screening as part of usual midwife and health visitor appointments (not standardised).

### Outcomes

#### Primary outcome: feasibility

The primary objective of the study is to evaluate feasibility of delivering a full-scale, single-blind, RCT within NHS services. In this feasibility trial, the a priori criteria for considering a full-scale RCT to be feasible are:

i. App engagement: overall completion rate of > 33% of daily app assessments AND > 50% of participants complete > 50% app-based EPDS assessments.
ii. Retention: > 80% participants retained at 8 weeks postpartum OR > 80% participants retained at 6 months postpartum.
iii. Data completeness: > 80% availability of relevant participant electronic health records (midwife, GP and health visitor electronic health records from baseline to 8 weeks postpartum) OR > 80% completion of the Mini International Neuropsychiatric Interview (MINI) at 6 months postpartum.

#### Justification of feasibility criteria

The first clause of the app engagement feasibility criterion (> 33% usage) is a threshold that is widely used in digital health studies to indicate sufficient app engagement, having originated in the Ecological Momentary Assessment literature [33]. In the current study, a participant meeting this threshold would answer app-based EPDS questions every three days, on average, providing plenty of mental health screening information (far more than is gathered in standard care). The second clause of the app engagement criterion (> 50% participants completing > 50% app assessments) was added to further strengthen the criterion by ensuring that usage was distributed across the sample rather than skewed towards a small number of high engagers.

Feasibility criteria ii and iii (retention and data completeness) each have two clauses, connected by ‘OR’. Two options are needed in each case because the primary outcome of the future full-scale RCT has not yet been decided. A secondary objective of the current feasibility trial is to examine suitability of two specific candidate primary outcomes for a full-scale RCT: (a) number of true positive and false positive screens identified by usual care (reported in electronic health records) between baseline and 8 weeks postpartum compared to those identified by digital screening in the same period, and (b) mental health status at 6 months postpartum (measured using the MINI). Depending on which primary outcome for the full-scale trial is chosen (a or b), retention at 8 weeks postpartum (for a) or 6 months postpartum (for b) is most relevant for feasibility criterion ii. Similarly, for feasibility criterion iii, availability of electronic health records (for a) or completion of MINI at 6 months postpartum (for b) would be most relevant. Hence, within the current feasibility RCT, it is necessary to examine retention at both timepoints and data completeness of two outcomes.

#### Secondary outcomes

Table 1 outlines the five secondary objectives and their associated outcomes.

**Table 1:** Secondary objectives and associated outcomes

	Objective	Outcome
1	To examine participant recruitment and identify barriers to recruitment	• The proportion of eligible individuals agreeing to participate • Reasons for declining to participate
2	To examine the suitability of two candidate primary outcomes for a full-scale trial	The extent and pattern of missing data, presence of floor/ceiling effects, and variability in responses in: a) number of true positive and false positive screens identified by usual care and digital screening between baseline and 8 weeks postpartum b) MINI at 6 months postpartum
3	To examine the suitability of other proposed clinical measures and health economic measures	The extent and pattern of missing data, presence of floor/ceiling effects, and variability in responses in: • PHQ-9 (baseline and after positive screen) • GAD-7 (baseline and after positive screen) • MINI (8 weeks) • Postpartum Bonding Questionnaire (8 weeks, 6 months) • Healthcare Resource Use Questionnaire (baseline, 8 weeks, 6 months) • EQ-5D-5L (baseline, 8 weeks, 6 months)
4	To examine the acceptability and usability of the digital screening system and participants’ experiences of using it	Qualitative (interview) and quantitative (MARS) data from participants in the app use group regarding app acceptability, usability, and experiences of using the digital screening system
5	To examine the safety of the digital monitoring system and study procedures	The number of adverse events (AEs) and serious adverse events (SAEs) and whether they are judged to be related to the app or other study procedures

#### Tertiary outcomes

- Summary statistics for the two candidate primary outcomes for a full-scale trial:
  - The number of true positive and false positive screens identified by usual care and digital screening between baseline and 8 weeks postpartum. True positives are defined as cases scoring above EPDS screening threshold (usual care threshold: total EPDS score ≥ 12; digital screening threshold: total EPDS ≥ 12 on three consecutive reports) and scoring ≥ 10 on PHQ-9 or GAD-7 in a subsequent assessment by a blinded researcher. False positives are cases scoring above EPDS screening threshold but scoring < 10 on PHQ-9 and GAD-7.
  - MINI score at 6 months postpartum.
- Summary statistics for the other clinical outcomes (MINI, Postpartum Bonding Questionnaire) and health economic outcomes (Healthcare Resource Use Questionnaire, EQ-5D-5L) at 8 weeks postpartum and 6 months postpartum.
- Key drivers of cost identified by the Healthcare Resource Use Questionnaire.

### Data collection and management

#### Overview

**Fig. 2:**
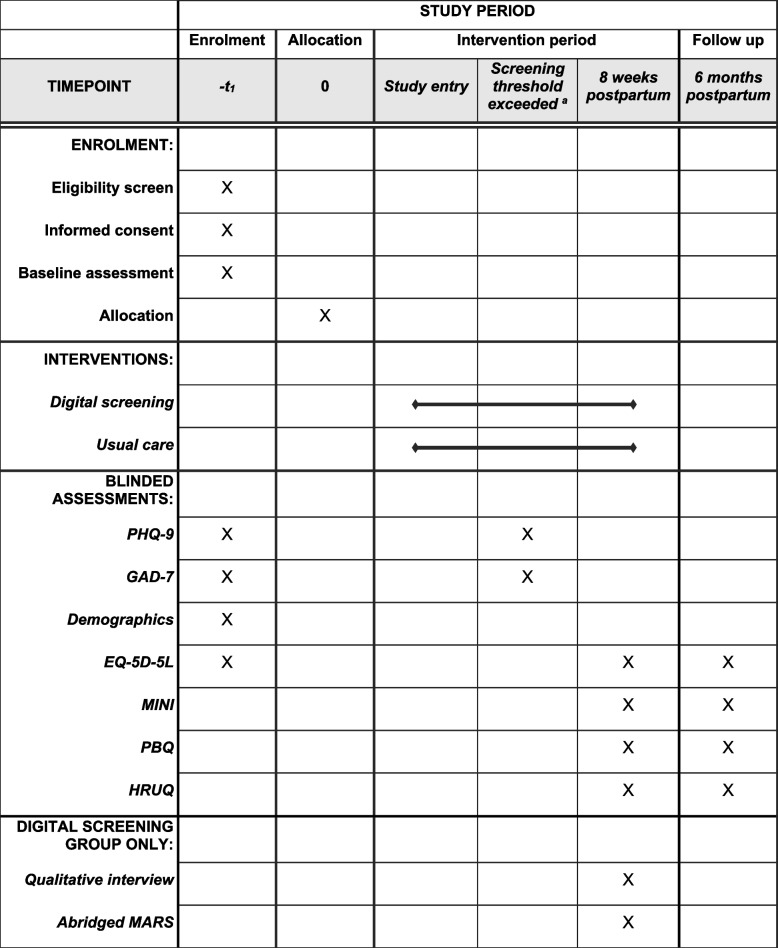
SPIRIT figure. ^***a***^If a participant scores above the screening threshold (via digital screening or usual care), blinded PHQ-9 and GAD-7 assessments will be completed to confirm whether it is a true positive screen. PHQ-9 = Patient Health Questionnaire; GAD-7 = Generalised Anxiety Disorder questionnaire; MINI = Mini International Neuropsychiatric Interview; PBQ = Postpartum Bonding Questionnaire; HRUQ = Healthcare Resource Use Questionnaire; Abridged MARS = Mobile App Rating Scale

Figure 1 outlines the study design and assessments, and Fig. 2 (SPIRIT figure) summarises assessment timing. Assessments will be conducted remotely (over the phone or via MS Teams) unless a face-to-face meeting is strongly preferred by the participant. The researcher will enter participant responses to study measures into an electronic case report form (e-CRF) via the web-based Qualtrics survey system. Participants will be reimbursed with £20 shopping voucher for each study assessment session they participate in.

#### Baseline assessment (both groups)

At baseline, a research assistant or research midwife will assess participants using three self-report measures: a 9-item measure of depression (PHQ-9, Patient Health Questionnaire) [30], a 7-item measure of anxiety (GAD-7, Generalised Anxiety Disorder questionnaire) [31] and a generic health status measure (EQ-5D-5L) [34]. Demographic information will be collected with a questionnaire assessing age, gender, ethnicity, employment status, family composition, native language and past psychiatric history.

#### Case note review (baseline until 8 weeks postpartum; both groups)

An unblinded researcher will review all participant electronic health records (midwife, GP, and health visitor case notes) on a weekly basis from baseline until 8 weeks postpartum and extract all reported EPDS scores (total score and individual item scores). Participants scoring above the standard care EPDS threshold (total EPDS ≥ 12) will be counted as a positive screen. Positive screens will be referred to a blinded researcher for assessment with PHQ-9/GAD-7 within 48 h. Participants scoring above threshold on the PHQ-9 (total score ≥ 10) [30] or GAD-7 (total score ≥ 10) [31] will be counted as true positives. Participants scoring below PHQ-9 and GAD-7 thresholds will be counted as false positives.

Additional cross-sectional information will be collected from electronic health records for all participants: answers to mental health screening questions and past psychiatric history reported at booking appointment, details of current childbirth (mode of delivery, live/still birth, any major obstetric complications), parity (total number of pregnancies reaching viable gestational age).

#### Blinded follow up assessments: 8 weeks and 6 months postpartum (both groups)

At 8 weeks and 6 months postpartum, a blinded researcher will assess all participants using the MINI, a short, structured diagnostic interview for DSM-IV and ICD- 10 psychiatric diagnoses [35]. Three relevant subscales of the MINI will be used in this study: major depression (subscale A), suicidality (subscale B) and generalised anxiety disorder (subscale N). Participants will also be assessed with three self-report measures during each follow-up assessment: a generic health status questionnaire (EQ-5D-5L), a bespoke questionnaire assessing health and social care resource use during the follow up period (unpublished), and a validated questionnaire assessing parental bonding with their baby (Postpartum Bonding Questionnaire) [36]. Regarding choice of assessment timepoints, the 8-week postpartum assessment aligns with the end of the intervention period. The 6-month postpartum assessment point was chosen to allow time for screening (digital screening or usual care screening) to have an effect on participants’ mental health. Specifically, time will be needed for participants screening positive for PND to be referred for additional mental health care (e.g. talking therapy, medication) and for that care to take effect.

#### App feedback: 8 weeks postpartum (unblinded assessment; digital screening group only)

An unblinded assessment will be conducted at 8 weeks postpartum to gather feedback about the study smartphone app. All participants from the digital screening group will be asked to give feedback on the app using the Abridged Mobile App Rating Scale (MARS), a 12-item questionnaire assessing acceptability and usability of health-related smartphone apps [37]. A sub-group of up to 30 participants from the digital screening group will be invited to take part in a semi-structured qualitative interview exploring the acceptability of digital screening and study procedures, and implementation barriers/facilitators. Participants will be purposively sampled to include individuals with a range of demographic characteristics and app-use levels (including app discontinuers). This will ensure that a variety of views are gathered regarding the acceptability of the app-based screening. Interviews will follow a topic guide (Supplementary material 1), last 30–60 min and be audio recorded using an encrypted device and later transcribed verbatim. Separate consent will be sought for qualitative interviews to be sure that participants fully agree to participate.

#### Data protection and confidentiality

Study data will be pseudonymised. All participants will be allocated a unique study ID number. Participant names and other identifying information will be removed from study data and interview transcripts, which will then be identified by the study ID number only. Personal identifiable data (name, address, audio-recordings, etc.) will be securely stored separately from pseudonymised study data. Confidentiality of information provided during the research will only be broken if a participant is assessed to be at risk to themselves or others (or discloses significant bad practice); participants will be informed of this procedure prior to giving consent.

### Data analysis

Analysis will be on an intention-to-treat basis, with participant data analysed according to the arm to which they were randomly allocated. Protocol deviations will be noted, and their frequency used to inform the power calculation for the larger study (c.f. contamination). A protocol deviation is defined by the study sponsor as “an action or incident inconsistent with the original, approved protocol that is documented after it occurs”. A CONSORT diagram will present information on the number of potential participants identified, number eligible/ineligible (with reasons for ineligibility), number declining to participate (with reasons, where reported), as well as information on the number of actual participants consented, baseline assessed, randomised, and retained in the study at both 8 weeks and 6 months postpartum. Additionally, the CONSORT diagram will detail the number of participants receiving the treatment to which they were randomised, withdrawals from the digital screening intervention arm (with reasons) and number of withdrawals/dropouts from the trial (by trial arm, with reasons).

#### Primary outcome

The key measures of feasibility will be reported using descriptive statistics:

i. App engagement: overall percentage of app-based assessments completed during the app-use period; percentage of participants completing > 50% app-based assessments.
ii. Retention: percentage of participants retained at 8 weeks postpartum; percentage of participants retained at 6 months postpartum.
iii. Data completeness: the extent and pattern of missing data from participant electronic health records (midwife, GP and health visitor records from baseline to 8 weeks postpartum) and MINI at 6 months postpartum.

#### Secondary outcomes

Appropriate descriptive statistics will be used to summarise: baseline participant socio-demographic data (by trial arm), the extent and pattern of missing data for clinical outcomes (PHQ-9, GAD-7, MINI, Postpartum Bonding Questionnaire) and health economics outcomes (Healthcare Resource Use Questionnaire, EQ-5D-5L) at each time point, questionnaire completion rates (i.e. the number of individual items completed), variability in the responses across participants and floor/ceiling effects, and the number and type of Adverse Events (by trial arm).

Data on the acceptability and usage of ClinTouch PND app will be analysed for the digital screening group only. Total and item scores on the MARS questionnaire will be summarised descriptively (median and range). Qualitative interviews with those selected in the app group will be analysed using framework analysis [38], with analysis managed using Nvivo software [39]. Framework analysis is a systematic qualitative research method which follows a structured approach, enabling researchers to identify key themes while maintaining transparency and rigor [38]. Its matrix-based structure facilitates comparison across cases and themes, ensuring coherence and depth in qualitative inquiry. The process involves several stages: familiarisation with the data, developing a thematic framework, indexing (systematically coding all transcripts), charting (creating the framework matrix table), and mapping and interpreting the data. Unlike purely inductive qualitative methods, framework analysis allows for both deductive and inductive coding [38], making it adaptable for studies wishing to explore predefined questions (e.g. “Is the frequency of digital screening (daily) suitable?”) as well as exploring unanticipated aspects of the qualitative data.

#### Tertiary outcomes

Summary statistics will be reported for each clinical outcome measure and health economics measure, by group, at each time point. As this is a feasibility study, no formal statistical tests comparing treatment groups will be conducted. No model-based imputation (e.g. multiple imputation) of missing data items will be attempted; missing data items will assume the mean value of all completed items on that measure for a particular individual (assuming that sufficient items have been completed). Health utility values will be estimated using the method recommended by NICE at the time of the analysis. Mean utility values will be reported at each time point and quality-adjusted life years (QALYs) estimated across the whole study period. These will be reported by treatment allocation group. Responses on the EQ-5D-5L will be compared with results from key clinical outcome measures to determine whether the characteristics (e.g. direction) of any observed health effects are comparable according to the different measures. The number and proportion of participants reporting use of different health and social care services (and quantity of services used) will be summarised by treatment allocation group.

### Data monitoring and auditing

#### Audit

The study will be subject to the audit and monitoring regime of the Greater Manchester Mental Health NHS Foundation Trust, the University of Manchester, Manchester University NHS Foundation Trust and Mersey and West Lancashire Teaching Hospitals NHS Trust.

#### Independent oversight

Independent oversight will be provided by a Trial Steering Committee (TSC), consisting of an independent chair, a statistician, a clinical academic, a parent representative, and the study Chief Investigator. All TSC members except the Chief Investigator are independent of the study team. The TSC will meet every 9 months to provide advice on the conduct, progress and safety of the research and to ensure that the project is conducted to the rigorous standards set out in the Department of Health’s Research Governance Framework for Health and Social Care [26] and the Guidelines for Good Clinical Practice [41].

#### Harms

A Standard Operating Procedure (SOP) will be followed to ensure that adverse events are monitored and reported throughout the study in line with sponsor policies and local site reporting systems. During this study, information about adverse events may be reported during study assessments, via spontaneous participant report, during safety phone calls (app group only), or may be gathered from electronic health records. All adverse events will be documented and assessed for seriousness, relatedness, and expectedness. Where needed, additional information will be sought to allow accurate categorisation. All events categorised as serious (Serious Adverse Events, SAEs) will be reported to the study sponsor and TSC chair. All adverse events categorised as serious, related to the study procedures, and unexpected (Suspected Unexpected Serious Adverse Reactions, SUSARs) will be reported to the study sponsor, TSC chair and ethics committee within seven calendar days.

### Patient and public involvement (PPI)

Throughout the study lifecycle, we have consulted and involved a diverse group of parents with relevant lived experience. An individual with lived experience of perinatal mental health problems is a co-investigator on the study and is actively involved in managing and delivering the research. We established a study PPI group whose members have varied experiences of parenting and perinatal mental health, including lived experience of postnatal depression, postpartum psychosis, and coping with stillbirth. The group is ethnically/linguistically diverse and includes Urdu, Cantonese and Arabic speakers, to reflect the languages in which the CareLoop PND app is available. The PPI group have had a significant impact on the study to date: providing detailed feedback on the design of the CareLoop PND digital screening system during a series of co-design workshops, designing and amending participant-facing documents (e.g. participant information sheets, study posters) to improve readability and presentation, filming and editing participant-facing videos, troubleshooting specific aspects of study procedures (e.g. how to maximise recruitment), helping to format documents translated into the minority languages (Arabic, Urdu, Cantonese), and taking part in role plays of study assessments as part of research staff training. The PPI group will continue to meet every 2–3 months during the study to oversee the study from a lived-experience perspective and to advise on emerging issues. Regarding wider stakeholder involvement, health professionals also contributed to the digital screening system design within two co-design workshops.

### Dissemination

Findings will be presented at relevant academic conferences and published in peer-reviewed academic journals. A lay summary of findings will be co-produced with the study Patient and Public Involvement group and distributed to any study participants who have indicated that they wish to receive it. The lay summary will also be disseminated via local community groups, NHS services and via the study website and social media channels. Study findings will be shared at locally at existing and bespoke public engagement events.

## Discussion

We developed a digital screening system (CareLoop PND) which uses a validated screening measure to identify individuals experiencing depression during the early postnatal period. Our proof-of-concept study showed promising results but, prior to implementation in NHS services, a full-scale RCT is needed to determine efficacy and cost-effectiveness. The current study aims to gather information required to plan and deliver a future full-scale RCT evaluating the CareLoop PND digital screening system. As such, this study is part of an overarching programme of work that has the potential to transform existing PND screening and to increase referral to perinatal mental health care, supporting the NHS Long Term Plan [8] ambitions.

This work is in line with recommendations of the Topol Review (“Preparing the healthcare workforce to deliver the digital future”) [42] regarding the strategic direction of the NHS to be a world leader in digital healthcare technologies. In particular, the Topol Review emphasises that “The NHS should expand research and development programmes, working closely with patients to co-create digital technologies and ensure that emerging technologies meet their needs.” [42, pg. 15]. The current study reflects these priorities by extensively involving patients throughout the research lifecycle, both in the overall design and management of the research and specifically in co-designing the CareLoop PND digital screening system. Further, by gathering in-depth qualitative information from participants regarding their experiences of and views about the digital screening system, we will explore the extent to which the current version of the system meets their needs, and what changes are needed to improve this in future iterations. Likewise, alongside the feasibility trial outlined in the current manuscript, we have conducted qualitative interviews with a range of healthcare professionals working with patients during the perinatal period (not yet published). Gathering detailed staff perspectives about digital PND screening will allow us to consider likely barriers and facilitators of eventual implementation in health services, to inform future implementation strategies.

In conclusion, we anticipate that the current study, and our wider programme of work, will contribute to the bank of evidence that will drive the future of digital NHS care. By identifying PND early and allowing prompt treatment, digital screening is likely to reduce the long-term negative impacts and costs of PND for parents, their children and health and social care services.

## Supplementary Information

Additional file 1. Qualitative interview topic guide for parents.

## Data Availability

Not applicable.

## Abbreviations

AE: Adverse event
EPDS: Edinburgh Postnatal Depression Scale
GAD-7: Generalised Anxiety Disorder questionnaire- 7
MARS: Abridged Mobile App Rating Scale
MFT: Manchester University NHS Foundation Trust
MINI: Mini-International Neuropsychiatric Interview
MWL: Mersey and West Lancashire Teaching Hospitals NHS Trust
NHS: National Health Service
NICE: National Institute for Health and Care Excellence
PHQ-9: Patient Health Questionnaire- 9
PND: Postnatal depression
PPI: Patient and Public Involvement
QALY: Quality-adjusted life year
RCT: Randomised Controlled Trial
SAE: Serious adverse event
SOP: Standard Operating Procedure
SUSAR: Suspected Unexpected Serious Adverse Reaction
TSC: Trial Steering Committee
