# Supplementary material for: Digital Assessment of Wellbeing in New Parents (DAWN-P): protocol of a randomised feasibility trial comparing digital screening for maternal postnatal depression with usual care screening
Source: Pilot Feasibility Stud. 2025 Apr 12;11:47. doi: 10.1186/s40814-025-01631-7 (PMC11992709; doi:10.1186/s40814-025-01631-7)
Supplement: Supplementary file 1 — Additional file 1. Qualitative interview topic guide for parents. [file 40814_2025_1631_MOESM1_ESM.docx]

**Qualitative interview topic guide for parents**

1. **Using the phone app**

1.1. Could you tell me about your experience of using the phone app?

1.2. What did you think about the way the questions were asked/presented in the app?

1.3. What do you think about the way the app looks?

1.4. How did you find the length of the assessments? (How long did it take you to complete each one?)

1.5. You’ve used the app for 8 weeks – what was it like using it for this long? How would you feel about using it for longer?

1.6. How did using the app fit in with your routine? (When did you answer the questions each day?)

1.7. What do you think about the length of time that the questions are available for? When did you tend to answer the app questions? When the phone first alerted you, or later?

1.8. Can you think of anything you would change about the app if you could? Could you tell me a bit about that please?

1.9. Did anything worry you about using the app? What in particular?

1.10. Was there anything about the app that you found particularly useful?

1.11. How would you feel about an app like this being used as part of standard postnatal care?

1.12. Is there anything else you’d like to tell me about using the app? (Do you have any more feedback that could help us improve the app?)

1.13. Would you recommend the app to a friend or family member? (Why/why not?)

1. **Study participation**

2.1. What were your reasons for taking part?

2.2. Could you tell me a bit about what you enjoyed about taking part in the study?

2.3. Was there anything that you didn’t enjoy? Can you tell me a bit about that?

2.4. Did you feel you got enough support from the researchers during the study? Is there anything else that would have helped?

2.5. How did you feel about getting phone calls from the researcher as well as using the app?

2.6. As the whole study was conducted remotely you didn’t meet the researcher face-to-face at any point. How did you feel about this? Do you feel like it interfered with your participation? In what ways?

2.7. Is there anything different we could have done to make the study run more smoothly?

2.8. Could you tell me a bit about why you weren’t able to continue completing the assessments? [if applicable]

2.9. Is there anything that would have helped you to continue? [if applicable]

2.10. Is there anything else you’d like to tell me about taking part in the study?
